# Configuration and rapid start-up of a novel combined microbial electrolytic process treating fecal sewage

**DOI:** 10.1016/j.scitotenv.2019.135986

**Published:** 2020-02-25

**Authors:** Hongbo Liu, Yicheng Lv, Suyun Xu, Zhongbing Chen, Eric Lichtfouse

**Affiliations:** a School of Environment and Architecture, University of Shanghai for Science and Technology, 516 Jungong Road, 200093 Shanghai, China; b Faculty of Environmental Sciences, Czech University of Life Sciences Prague, Kamýcká 129, 16500 Prague, Czech Republic; c Aix-Marseille Univ, CNRS, IRD, INRA, Coll France, CEREGE, 13100 Aix en Provence, France

**Keywords:** Coupled ABR-MFC-MEC, Fecal sewage treatment, Rapid start-up, External voltage, Biological analysis

## Abstract

Most of the developing countries are in need of sanitary toilets due to insufficient supporting facilities and proven technology mainly on disposal of fecal sewage. A microbial fuel cell (MFC)-microbial electrolytic cell (MEC) coupling with an anaerobic baffle reactor (ABR) was used to realize simultaneous removal of nitrogen and carbon in fecal sewage and complete energy recycling. Configuration and rapid start-up of the ABR-MFC-MEC process treating fecal sewage was systematically studied. Results showed that the application of an external voltage of 0.5 V can shorten the start-up time and improve hydrogen production rate to 3.42 × 10^−3^ m^3^-H_2_/m^3^/d in the MEC unit, where the double-chamber MFC can drive MEC completing the synchronous coupling start-up. In the single and double chamber systems, bio-electrochemical processes both enhanced shock resistance capacity of the whole ABR-MFC-MEC process during coupled operation, with chemical oxygen demand (COD) removal rates of 99.2% and 98.9% for the single and double chamber systems respectively. Based on results of biological analysis, the coupled system has a distinct selective effect on microbial population and each unit has high microbial diversity to enhance the stability and resistance of the whole system for treatment of feces and urine.

## Highlights

• A novel coupled bio-electrochemical process was developed to treat fecal sewage.
• Optimal configuration and start-up mode of the coupled process were determined.
• 0.5 V was determined as the threshold voltage shortening start-up time.
• The coupling mechanism of the combined process were investigated.

## Graphical abstract

**Figure:**
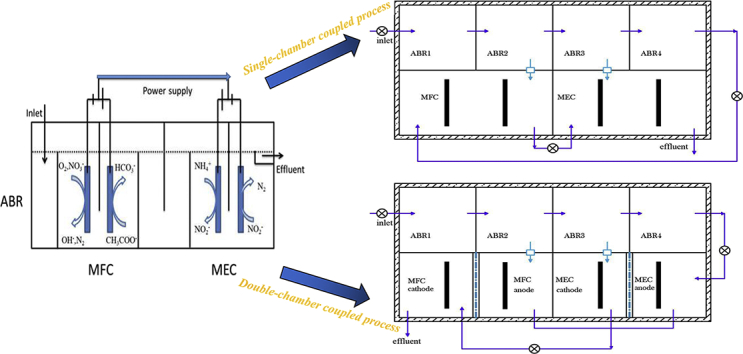

## 1 Introduction

Most of the developing countries are in need of sanitary toilets due to insufficient supporting facilities and proven technologies, especially in the rural areas (Leuzzi, 2012). In recent years, the Chinese government advocates the construction and renovation of hygienic toilets to improve the human settlement. The vital part of the development of the modernization of sanitary rural toilets is treating feces and fecal sewage since the untimely and incompleteness of the feces and fecal sewage disposal easily results in surface water and soil contamination (Michael and Charlene, 2008).

Toilet fecal sewage is known as black water, which refers to general term of urine, feces and flushing water. In domestic sewage, 51% of chemical oxygen demand (COD), 91% of nitrogen (N) and 78% of phosphorus (P) come from black water (Sari et al., 2007). Furthermore, black water also contains a large number of pathogenic bacteria, organic compounds and ammonia (NH_4_^+^-N) that can seriously damage land and water resources (Hertel et al., 2015; Mawioo et al., 2016). Pathogens in feces can also be enriched in animals and plants and enter human body through the food chain (Li-Jie and Wei, 2003). However, it is inefficient and risky to treat fecal sewage by traditional methods such as septic-tanks. The reason is that feces and fecal sewage in the septic-tank can permeate into the soil in the long run, which will lead to the pathogen pollution of the soil; septic-tanks also have low volumetric load and digestive efficiency, long hydraulic retention time (Paulo et al., 2013).

Nowadays, fecal sewage treatment technologies are continuously developing toward low sludge yields, heavy sludge concentration and high load (Glassmeyer et al., 2005). Energy production and nutrient recovery is receiving more and more attention when treating urine and feces; while bio-electrochemical technologies present low sludge yields and energy self-sufficiency (Badea et al., 2019; Hong and Logan, 2004; Ren et al., 2012). Bio-electrochemical processes such as microbial fuel cell (MFC) and microbial electrolysis cell (MEC) have been confirmed to recover energy and nutrition from fecal and urine (Aqaneghad and Moussavi, 2016). MFCs have the ability to generate energy by human feces or urine as the feedstock, and synchronously recover nitrogen, phosphorus and potassium from urine (Chouler et al., 2016; Du et al., 2011; Kuntke et al., 2012). MECs processes harvested hydrogen gas in the cathode using various types of biomass and ammonium while degrading pollutants with the stimulation of electric current (Chouler et al., 2016; Du et al., 2011; Kuntke et al., 2014; Kuntke et al., 2012; Liu et al., 2017a).

The ABR-MFC-MEC system can be interpreted as an electrochemical microbial reactor coupling with an anaerobic baffle reactor (ABR), where the ABR unit functionalizes as the anaerobic fermentation reactor decomposing macromolecular organic matters from the fecal sewage. The electrical bacteria in MFC-MEC units further degrade micro-molecule organic matters and convert the chemical energy into electric energy (Leu and Wu, 2014). Moreover, microbial current from MFC could drive MEC to degrade organic acids, while the surplus microbial current can stimulate and strengthen microbial metabolism in the system (Kang et al., 2012). Meanwhile, the coupling of bio-electrochemical system (BES) could promote the transformation of the residual volatile fatty acids (VFA_S_) in the effluent of ABR unit, thus greatly improve the anaerobic digestion rate of the overall process. In addition, hydrogen produced by the MEC unit is helpful for hydrotrophic methanogens (Guo et al., 2010).

Bio-electrochemical coupling system presents obvious superiority over the traditional denitrification process (Bo et al., 2014). The electrochemical functional bacteria enriched on the MEC anode could utilize ammonia as electron and be oxidized to nitrite as depicted in Eq. (1); some researches also indicated that ammonia might be anaerobically oxidized to nitrate using MEC anode as the electorn acceptor (Bo et al., 2014); while the anodes of MFC and MEC degraded the organic matters from the effluent of ABR by capturing electronic from external circuit to the cathode as shown in Eq. (2). The autotrophic denitrifying bacteria enriched on bio-cathodes could accept electron for completing the nitrite or nitrate reduction, thus to strengthen the denitrification process as shown in Eqs. (3), (4) (Zhen et al., 2009). In addition, the autohydrogenotrophic denitrifying bacteria enriched on bio-cathodes can also utilize hydrogen produced by the MEC cathodic reaction to reduce nitrate (Prosnansky et al., 2002). Moreover carbon sources can be more concentrated in the DeNOx reaction under stimulation of an electric field (Sakakibara and Kuroda, 2010). Thus, the TN removal rate of the whole system is enhanced without input of additional carbon source.

(1) $$\text{Anode}\;\mathrm{MEC}:2NH_{4}^{+}+2H_{2}\to NO_{2}^{-}+6e^{-}+8H^{+}$$

(2) $$\text{Anode}\;\mathrm{MFC}/\mathrm{MEC}:CH_{3}\mathrm{CO}O^{-}+4H_{2}O\to 2\mathrm{HC}O_{3}^{-}+9H^{+}+8e^{-}$$

(3) $$\text{Cathode}\;\mathrm{MFC}/\mathrm{MEC}:2NO_{2}^{-}+6e^{-}+8H^{+}\to N_{2}+4H_{2}O\;$$

(4) $$\text{Cathode}\;\mathrm{MFC}/\mathrm{MEC}:2NO_{3}^{-}+10e^{-}+12H^{+}\to N_{2}+6H_{2}O$$

Our previous study had confirmed that the coupled ABR-MFC-MEC system can remove carbon and nitrogen from the fecal sewage efficiently (Liu et al., 2017b). Under low electric field, electron transfer promotes solid organic matter hydrolysis while the ABR baffle can block most suspended solids in black water and reduce sludge production (Park et al., 2017). The baffle increases hydraulic residence time (HRT), and separates the hydrolysis acidification stage and methane generation stage in space, which improves the efficiency of anaerobic digestion. Moreover, the coupled system ensure that each chamber could form their own dominant bacteria in the operation stage and establish collaborative symbiosis while jointly promoting the simultaneous removal of nitrogen and carbon in black water in the coupled system (Zhang et al., 2011).

Although the coupled system is very effective in terms of COD and NH_4_^+^-N removal from low load fecal sewage, the start-up process and its coupled characteristics were not well explained. Therefore, this study investigated the configuration and start-up mode of the ABR-MFC-MEC system, aiming at optimizing the start-up process of the ABR-MFC-MEC system, examining the performance of the single and double chamber ABR-MFC-MEC processes at a high load operation, and further exploring the coupling mechanism of bio-electrochemical fecal sewage treatment by biological community diversity analysis.

## 2 Materials and methods

### 2.1 Experimental device

**Fig. 1:**
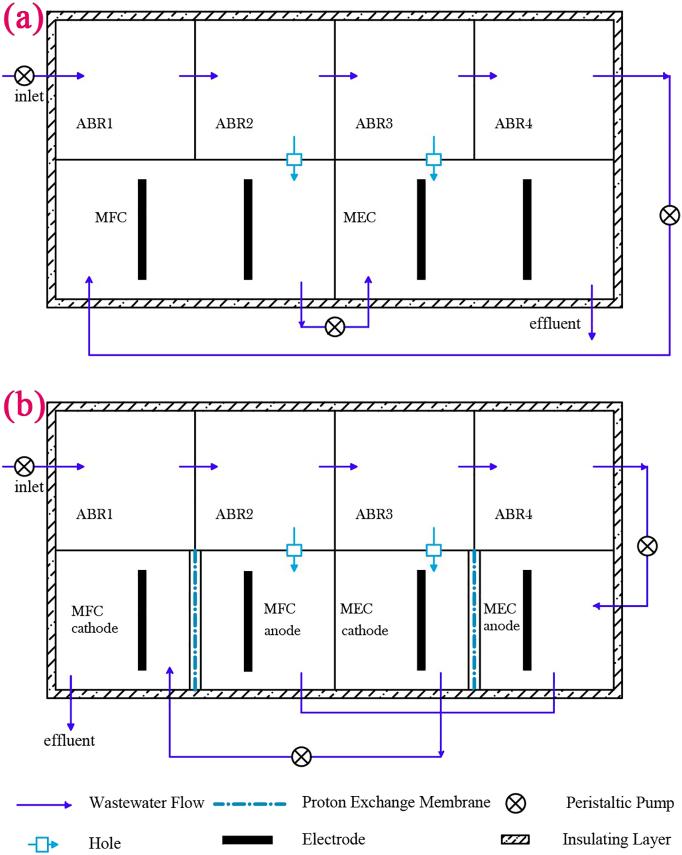
Diagram of the single-chamber ABR-MFC-MEC reactor (a); diagram of the double-chamber ABR-MFC-MEC reactor (b).

Diagram of the single-chamber and the double-chamber ABR-MFC-MEC reactor are illustrated as Fig. 1 (a) and Fig. 1 (b), respectively. The single and double chamber reactors are made of plexiglass, the effective volume both are 28 L, with the length, width and height of 640 mm × 180 mm × 250 mm respectively. The ABR reactor has 4 chambers; each chamber is constituted of up-flow-module with an angle of 45° to the baffled plate and a down-flow chamber, and on the upper part of each chamber with air vents. Anode and cathode of the MFC reactors are graphite carbon felt and wire mesh respectively with the same size of 8 cm × 10 cm. The effective volume of the single-chamber MFC is 9.6 L; while both the anode and cathode chambers of the two-chamber MFC are 4.8 L; microporous aerators are set at the bottom of both the cathode chamber of the double-chamber MFC and single-chamber MFC. Total volume of the MEC unit equals to the MFC unit using graphite carbon felt (8 cm × 10 cm) for both anode and cathode. The anode and cathode are separated by Nafion117 proton exchange membrane (thickness 50.8 μm, weight 100 g/m^2^) in the two-chamber MEC; while the MEC are connected with MFC through wires to realize the transfer and effective utilization of electric energy with a diode connected between two electrodes to control the current direction from MFC to MEC. The temperature of the single and double chamber reactors are remained constant by temperature controller (PCI-6221, USA), and water heating is in the insulation layer to carry out the temperature of the reactors at 32.0 ± 0.5 °C.

As shown in Fig. 1 (a), in the single-chamber reactor, the black water is fed into the ABR1 at a constant rate with the feed pump and flows into ABR2-ABR4 in turn through the baffled plates, the black water in the ABR2 and ABR3 units flow through the holes to the MFC and MEC reactors; the treated black water in the MFC unit is sent to the MEC unit by the peristaltic pump. As shown in Fig. 1(b), the flow pattern of double-chamber and single-chamber reactors are almost identical, with the differences lie in the fact that black water in the ABR2 and ABR3 units flow through the holes to the anode chamber of MFC and the cathode chamber of MEC, while the treated black water in ABR4 is pumped into the anode chamber of MEC.

### 2.2 Wastewater and inoculated sludge

Both real and synthetic black water were used in the study. Synthetic black water was used in the start-up stage while the real fecal black water was used in the later operation with COD_Cr_ 1200–1500 mg/L, BOD_5_ 500–800 mg/L, NH_4_^+^-N 120 mg/L，PO_4_^3−^-P 8–15 mg/L and pH 6–9. The synthetic black water consists of NH_4_Cl 0.382 g/L, CH_3_COONa 1.60 g/L, C_6_H_12_O_6_ 0.7 g/L, KH_2_PO_4_ 0.01 g/L, MgSO_4_·2H_2_O 0.01 g/L, NaHCO_3_ 0.1 g/L and 1 mL/L trace element; the initial pH of the influent synthetic black water is adjusted to 7.2. The real black water was taken from the small storage tank for an ecological toilet in Shanghai. The anaerobic flocculent sludge for the experiment was taken from the sludge thickener in Shanghai Dongqu sewage treatment plant. The sludge was anaerobic acclimatized for 1 month in advance with synthetic blackwater as nutrient solution before it was respectively inoculated into the ABR, MFC and MEC reactors according to the volume ratio of sludge to water 1:4 with a mixed liquid suspended solid concentration of 4.7 ± 0.3 g/L, and a volatile organic matter content of about 33%.

### 2.3 Operating conditions

#### 2.3.1 Start-up of the ABR unit

The mode of circulated intermittent (peristaltic pump to send the ABR4 effluent to ABR1) and gradually increasing COD load was taken for start-up of the ABR reactor at 32.0 ± 0.5 °C while fixing 7 days as a cycle to renew synthetic black water. The ABR unit started with a low load with the influent COD concentration kept at 200 mg/L and the NH_4_^+^-N concentration throughout maintained around 100 ± 5 mg/L. When the COD removal rate reached over 50%, elevated the COD concentration to 900 mg/L; continually elevated the COD load to 1600 mg/L when the COD removal rate reached over 60%. Successful start-up of the reactor was achieved when the COD removal rate maintained over 81.2% within the cycle (the influent COD was 1600 mg/L).

We inoculated the same sludge and introduced the synthetic black water to two ABR reactors in parallel, in order to explore the effect of temperature and nitrate on the start-up of ABR; the experiment adopted the intermittent mode with 6 days as a cycle. The reactor was initially maintained at 32.0 ± 0.5 °C by the insulation mode described in Section 2.1; after ran 2 cycles at 32.0 °C, decreased the temperature to 19 °C ± 0.5 °C, and after 2 cycles to 5 °C ± 1 °C by moving the reactor outside in December. To study the influence of nitrate concentration on the start-up of ABR at 32.0 ± 0.5 °C, the influent nitrate concentration increased from 0 mg/L to 30 mg/L and then to 60 mg/L (ran 2 cycles at each concentration).

#### 2.3.2 Start-up of MEC and MFC units

The individual start-up of MFC and MEC units were started through sequential batches at 32.0 ± 0.5 °C. The single and double chamber MFC were micro-aerated with an intensity of 1.875 m^3^ –O_2_/m^2^·h near the bio-cathode during the start-up stage; the influent COD was 1200 mg/L. A 1200-Ω external resistance was connected with the MFC unit and continuously recorded the voltage change of the resistance by a high resolution analogue-to-digital data logger (Pico Technology, UK). A DC power supply (IT8800 series) was used to power the MEC with 1 Ω resistance during the start-up stage of the MEC unit. Within four cycles the external voltage was improved step by step from 0.1 V to 0.7 V.

#### 2.3.3 Operation of the coupled ABR-MFC-MEC process

After individual units were successful started up, the single and double chamber ABR-MFC-MEC processes were combined to treat fecal sewage with continuous-flow with aeration at 1.875 m^3^ O_2_/m^2^·h near the MFC cathode, where NH_4_^+^-N concentration = 110 ± 5 mg/L, HRT = 1 d and temperature was 32.0 ± 0.5 °C.

### 2.4 Analytical methods

COD, NH_4_^+^-N, total nitrogen (TN), total phosphorus (TP), and sulfate (SO_4_^2−^) were analyzed with standard methods (American Public Health Association 1998). Water temperature and pH were measured by a thermometer (Leichi, China) and the phs-3c pH meter (Essence, China) respectively. Dissolved oxygen (DO) was analyzed by an analyzer (JPB-607A). Gas phase from the bioreactors was analyzed by a gas chromatography (Agilent Technologies 6890N).

At the end of each operational run, the biomass samples from each chamber and the graphite felt anode were collected to conduct microbial community analysis by high-throughput pyrosequencing on an Illumina platform (IlluminaMiseq PE 300). The total DNA of all samples were extracted by the PowerSoil™ DNA isolation kit (Mo Bio Laboratories Inc., Carlsbad, CA). The sequencing primers were 338F (5′-ACTCCTACGGGAGGCAGCAG-3′) and 806R (5′-GGA CTA CHV GGG TWT CTA AT-3′) for the relevant region of the 16S rRNA gene. The raw sequencing data were deposited into the NCBI database (PRJNA305812). Biodiversity index, species richness and similarity coefficient were calculated using the Majorbio I-Sanger Cloud Platform to compare the microbial diversity and richness between the samples.

## 3 Results and discussion

### 3.1 Rapid start-up of the coupled ABR-MFC-MEC process

#### 3.1.1 Start-up of the ABR unit

**Fig. 2:**
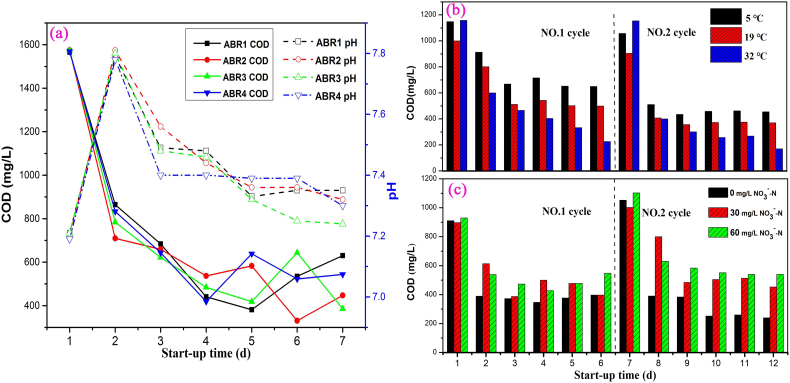
COD and pH start-up profiles in four chambers of the ABR unit (a); effects of temperature (b) and nitrate concentrations (c) on start-up of the ABR unit.

COD and pH profiles in four chambers of the ABR unit at the last cycle of the start-up period were illustrated in Fig. 2(a), with an initial COD concentration of 1600 mg/L. Concentration deviation of pollutants in four chambers (ABR1 - ABR4) of the anaerobic unit was relatively small due to intermittent internal circulation. Mutual separation characteristics of the chambers were not obvious, indicating that the four ABR chambers could be started simultaneously. The fastest organic compounds degradation occurred in the first two days, yielding COD removal rates of 44.8%, 55.0%, 50.2% and 46.9% in the four chambers respectively. The pH rose rapidly to about 7.8 in the second day with rapid COD degradation. Since the optimum pH of methanogens was about 7.0–7.2 and the higher pH inhibited the methanogenesis efficiency (Dawei et al., 2010), a slowed down COD degradation was observed in the following days. The baffles of ABR ensured the relative stability of sludge concentration in 4 chambers, so as to assure stable start-up of the system (Liu et al., 2018). Therefore, pH is an important limiting factor affecting reaction and methanogenesis of the system. Maintaining pH between 7.2 and 7.5 is critical for rapidly starting up of the ABR unit. The COD removal rate during a cycle could be stabilized at around 81.2% when COD load was 1600 mg/L implying successful start-up of the ABR unit. Thus the ABR unit can be started rapidly by increasing the influent load step by step in a short period.

To study the effect of temperature on rapid start-up of the ABR, the initial COD is set as 1050 ± 100 mg/L and the NH_4_^+^-N concentration maintained as100 ± 5 mg/L. In the first cycle, the COD removal rates were 43.4%, 50.0%, and 80.4% under 5.0 ± 1.0 °C, 19.0 ± 0.5 °C and 32.0 ± 0.5 °C, respectively (Fig. 2(b)). Temperature mainly affects the activity of dehydrogenase and urease (Rohwerder et al., 2003); low temperature may result in failure of ABR start-up. Effect of nitrate concentration on rapid start-up of the ABR unit was demonstrated with Fig. 2(c), under the initial COD concentration of 1000 ± 100 mg/L and the NH_4_^+^-N concentration 100 ± 5 mg/L. Fig. 2(c) illustrated that when nitrate exists in the start-up stage, COD degradation of the ABR unit will be inhibited (Xie et al., 2011). Nitrogen oxides also have a certain toxic function on methanogenic bacteria (Tugtas and Pavlostathis, 2010). Therefore, controlling the temperature at 32 °C and reducing nitrates content can enhance the COD removal rate and shorten ABR start-up time.

#### 3.1.2 Independent start-up of MEC and MFC units

**Fig. 3:**
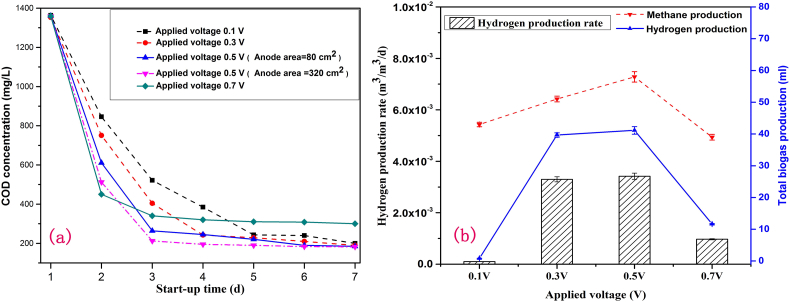
Effect of external power application intensity on substrate degradation of the MEC unit during the start-up stage (a); Effect of voltage on total biogas production and hydrogen production rate of the MEC unit within a cycle (b).

The MEC unit could be considered as successfully started when the biogas yield and/or the substrates degradation on MEC anode tend to be stable. Fig. 3(a) indicated that comparing with 0.1 V and 0.3 V operations, the COD removal rate could reach 85.0% on the third day when an external power of 0.5 V is applied. This can accelerate the conductive bacteria enriched on the anode surface and accelerate the carbon sources consumption so that the start-up period is shortened. Electroactive bacteria can overcome higher thermodynamic energy barrier by increasing applied voltage and promoting the reaction rate closer to the thermodynamic equilibrium constant (K_C_ value). The raise of the applied voltage can also enhance the conductive bacteria activity and shorten the start-up cycle (Chae et al., 2008). Moreover, the bacteria attachment area became larger by increasing anode area to 320 cm^2^. The final organics removal rate decreased when the input voltage of MEC increased from 0.5 V to 0.7 V, indicating that the microbial activity and growth might be inhibited when the applied voltage exceeded threshold. The start-up of the MEC unit will be inhibited under excessive voltage over 0.7 V. Zhen (Zhen et al., 2017) indicated that higher input voltage on MEC might inhibit activities of electroactive bacteria, thus caused the upset and instability of the BES processes. Luo et al. (2005) implied that high levels of voltage could increase the surface hydrophobicity and resulted in the cell rupture of electroactive bacteria.

Fig. 3 (b) indicated that nearly no hydrogen generation was observed within a cycle when the external voltage application is 0.1 V. When the applied voltage increased to 0.5 V, the hydrogen production increased within a cycle, and hydrogen production rate improved to 3.42 × 10^−3^ m^3^-H_2_/m^3^/d. The hydrogen and methane yields simultaneously decreased when the input voltage increased from 0.5 V to 0.7 V, due to excessive current stimulation. Therefore, the optimum electrolysis voltage which can accelerate start-up of the MEC reactor is 0.5 V; while excessive or low electrolysis voltage may be detrimental to the start-up. Feng also found that the methane yield of the BES system was enhanced at 0.3 V and inhibited at applied voltages higher than 0.6 V and the hydrogen was produced at 0.6 V under the cost of less methane yield (Feng et al., 2015). In addition, the maximum methane production efficiency was only 5.9% at the start-up stage when the voltage was 0.5 V, due to partial competition of hydrogen production.

**Fig. 4:**
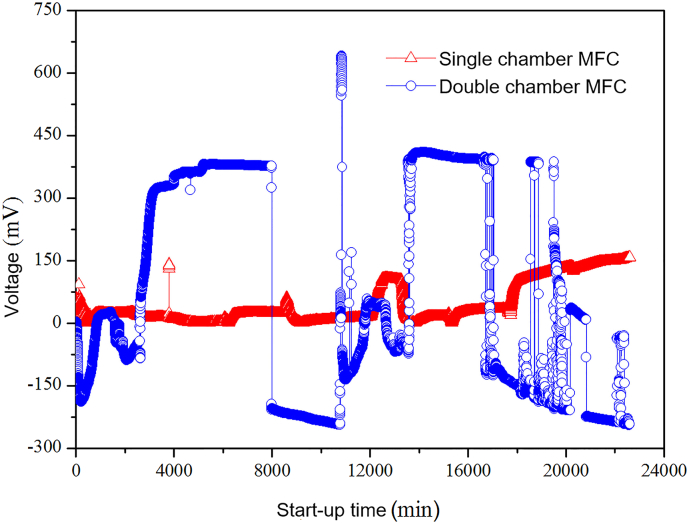
Voltage changes during start-up of the MFC unit with two configurations.

Microbial electricity generation profiles of both the single and double-chamber MFCs were demonstrated in Fig. 4, where the double-chamber MFC started faster with the rapid consumption of substrates. Output voltage of the double-chamber MFC can be rapidly stabilized above 400 mV with sufficient substrates, but the electrode reversal phenomenon of both anodes and cathodes occurs. Start-up of the single-chamber system comparing to the double-chamber system is slower, but the electrodes seldom reverse. The study of Lee found that the average voltage in double-chamber MFC were higher than in the single-chamber MFC under the same conditions, however the output voltage of double-chamber MFC is more unstable (Lee et al., 2016). The dissolved oxygen near the cathode that diffused to the anode in the single-chamber system could stimulate the rapid growth of non-electric microbes and consume organic substrates, led to electronic loss or low electricity production (Wang et al., 2014). Our previous study also revealed that increasing aeration intensity near the MFC cathode and the MFC anode area could improve output voltage (Liu et al., 2017b).

Starting voltage of the MEC unit should exceed 0.14 V to assist the electrons released from the electroactive bacteria of the anode (Parkhey and Gupta, 2016); thus the voltage generation of the double-chamber MFC unit can drive the MEC unit to complete synchronous coupling start-up. The single-chamber MFC and MEC completed individual start-up separately, and then instead of the DC power supply, the microbial current from the MFC unit was used as power supply for the single-chamber MEC. Finally, the MFC-MEC system was coupled with the ABR reactor to enhance performance of the ABR reactor.

### 3.2 Performance of the coupled ABR-MFC-MEC process

**Fig. 5:**
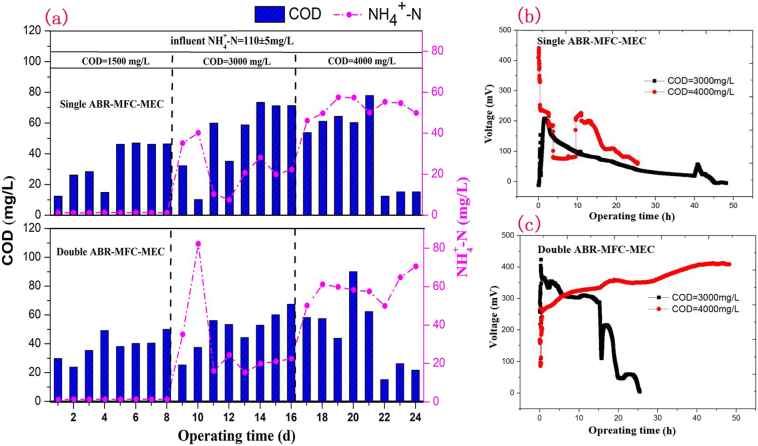
COD and NH_4_^+^-N removal in the single and double-chamber ABR-MFC-MEC processes (a); Electricity Production profiles of single-chamber (b) and double-chamber (c) ABR-MFC-MEC processes.

After successful start-up of the single and double chamber ABR-MFC-MEC processes, a continuous-flow operational mode was adopt to treat fecal sewage with different COD loads (Fig. 5). When the influent COD was elevated from 1500 ± 50 to 4000 ± 50 mg/L, both the single-chamber and double-chamber systems remained stable, with maximum COD removal rates of 99.2% and 98.9% respectively. Therefore stimulating the coupled BES system with a low current density could enhance shock resistance capacity of the whole process (Zhen et al., 2017). When influent COD in the first cycle was 1500 mg/L, the effluent ammonia of two systems remained at a very low level (about 1.3 mg/L); however, when the influent COD increased to 3000 mg/L and 4000 mg/L, the NH_4_^+^-N removal rates decreased to 51.7% and 46.4% respectively. On one hand the increase of influent COD concentration resulted in mass breeding of digestive bacteria, inhibited nitrifying bacteria in the systems (Chang et al., 2005); on the other hand the enhanced COD load may inhibit the anodic oxidation of ammonia in MEC unit (Virdis et al., 2008), leading to the decreased ammonia removal rate.

The electricity production profiles of the single and double-chamber processes at high COD loads were demonstrated in Fig. 4 (b)-(c). Compared with the single-chamber process, the double chamber coupled process can maintain higher output voltage. When the influent COD was 3000 mg/L, the highest output voltage is 400.0 ± 5.0 mV, and can maintain stable electricity production for 15 h before slowly decreased. When the influent COD was 4000 mg/L, the output voltage of the double-chamber process increased gradually with the increase of operating time. Therefore, the electrogenetic bacteria were more concentrated on the microbial anode to enhance power production process in the double-chamber ABR-MFC-MEC process.

### 3.3 Environmental implication from the microbial structural diversity analysis

Biomass samples from different locations of the combined ABR-MFC-MEC process have been taken to better understand microbiological characteristics and possible metabolic activities. Information of sampling locations, operating modes and sequences is shown in Table 1.

**Table 1:** Sludge sampling locations and sequence.

Sampling location	ABR1	ABR4	Single-MEC	Single-MFC	Double-MEC anode	Double-MFC anode
Operational mode	COD≤3000 mg/L；HRT = 12 h；C_sulfate_ ≤ 10 mg/L					
Sample ID	Sample I	Sample II	Sample III	Sample IV	Sample V	Sample VI
Sequences	60,116	33,138	62,899	69,005	65,199	71,199
Bases (bp)	26,370,211	14,638,885	27,787,896	30,339,643	28,819,923	31,310,751
Average (bp)	438.66	441.76	441.79	439.67	442.03	439.76

The Ace, the Chao, the Shannon and Simpson indexes respectively indicated the species richness and diversity of bacterial flora in each chamber. The samples were pretreated for OTUs clustering analysis before the diversity index is calculated as in Table 2. The Ace and Chao1 can also qualitatively estimate the total number of species in the system. The ACE and Chao1 indexes in ABR1 and ABR4 increased 3.2% and 6.6% respectively. For the single-chamber MEC to single-chamber MFC, the indexes of ACE and Chao1 decreased by 5.0% and 7.4% respectively. From the double-chamber MEC anode to the MFC anode, the indexes of ACE and Chao1 decreased by 1.5% and 1.6% respectively.

**Table 2:** Diversity index and population abundance index of sampling point (97% similarity nucleotide homology).

Sample ID	Sequence number	OTUs	ACE	Chao1	Simpson
ABR 1	53,626	1386	1487	1496	0.0098
ABR 4	28,773	1360	1535	1563	0.0199
Single-MEC	56,052	1487	1605	1654	0.0082
Single-MFC	63,072	1418	1524	1531	0.0077
Double-MEC anode	59,747	1305	1413	1445	0.0229
Double-MFC anode	67,157	1263	1391	1421	0.0482

Results of the above 4 indexes revealed that although ABR, MFC and MEC were provided with the same source of seeding sludge, microbial structure changed after operation for 3 months. The microbial diversity increased gradually and then decreased slowly with the direction of fecal sewage flow. High substrates concentration in ABR1 can enrich a large number of methanogens and anaerobic ammonia-oxidizing bacteria. The bacteria that can obtain energy with electron transfer were enriched on the anode electrode of MFC and MEC units while the bacteria that cannot deliver electrons were eliminated during the operation, promoting the centralization of microbial community structure in the systems. Moreover, the double-chamber configuration enhanced the dominance of the electrogenic bacteria. The Simpson index (also called the dominance index), reflecting the ratio of dominant microbial community biomass to total community biomass. Results of the Simpson index indicated that the dominant bacteria proportion in ABR4 was larger than that in ABR1. However, there are more predominant electro-genetic flora bacteria in the MFC anode chamber than in the MEC anode chamber of the double-chamber system.

**Fig. 6:**
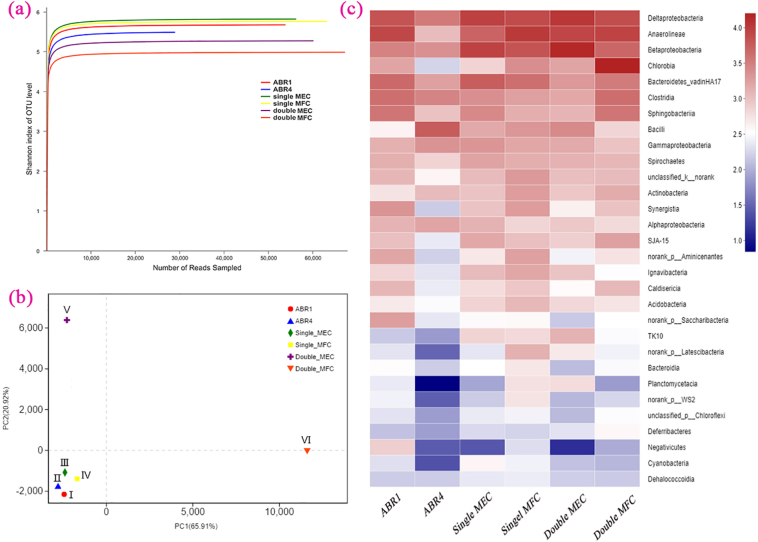
The curve of Shannon-Wiener rarefaction analysis based OTUs (a); PCoA of the microbial community structures (b); Heat map graph of similarity and difference of community distribution at class level (the Top 30 of relative abundance) (c).

The Shannon-Wiener curves (based on OTUs) were also used to reflect the variation of communities' species diversity; the larger the index, the higher the complexity of the communities (Gray, 2000). In Fig. 6(a), all the Shannon values of random samples were larger than 4. The Shannon values of the double-chamber MFC (anode sludge) was the smallest, indicating that the electro-genetic bacteria were more concentrated to enhance power generation process. Rabary et al. also indicated that MFC could has an intense selection effect on microflora (Rabaey et al., 2004).

Principal Coordinates Analysis (PCoA) results in Fig. 6(b) reveal that the double chamber of MEC and MFC (Sample V, VI) and ABR (Sample I, II) communities are obviously separated despite their same origin; while the single-chamber coupled system (Sample I, II, III, IV) communities are closer to each other. There are more independent microbial species in each chamber of the double-chamber system. The heat map graph of community structures at class level in Fig. 6(c) shows that the species distribution of different units shows great difference. The main species differential lie in Deltaproteobacteria (δ-Proteobacteria), Betaproteobacteria (β-Proteobacteria), Chlorobia, Clostridia, Bacilli and Bacteroidetes vadinHA17. The δ-Proteobacteria and β-Proteobacteria abundance increased near the bio-electrochemical anode, which is the main electro-genetic bacteria in coupled systems (Chaudhuri and Lovley, 2003; Fedorovich et al., 2009). Chlorobia as non-oxygen-producing photosynthetic bacteria has been abundantly enriched near the MFCs anode, suggesting that it can adapt to power generation process of the MFC unit (Rubaba et al., 2013). The class of Clostridia, Bacteroidetes and Bacilli, the confirmed electrogenesis bacteria (Schamphelaire et al., 2010), are also abundant near the MFC and MEC anodes of our study. The coupled system has a distinct selective effect on microbial structure.

The ABR-MFC-MEC system achieves bacterial flora with different functions to coexist synergistically and vie with each other. Generally, the increase of microbial diversity is in favor of resisting toxicity and other hostile environment (Zhao et al., 2016) and thus enhance the stability of the whole system, orderly completed the four processes of anaerobic methanation, electricity generation, simultaneous decarbonization and carbon removal and microbial electric stimulation. Therefore, the coupled system is able to handle high-polluted fecal sewage and is in line with the idea of the development of ecological toilets (Kumwenda et al., 2017).

## 4 Conclusions

Start-up time of the ABR unit can be shortened by controlling the nitrate concentration in influent and maintaining temperature at 32 °C with pH in the range of 7.2–7.5; meanwhile the MFC configuration has significant influence on its electricity generation. The application of an external voltage can shorten the start-up time and increase hydrogen production of the MEC unit, where the double-chamber MFC can drive MEC to complete synchronous coupling start-up. Both the single-chamber and double-chamber systems perform well during the operation stage, with the COD removal rates at 99.2% and 99.0% respectively. However, higher COD loads inhibited ammonia removal in single-chamber and double-chamber systems. The coupled system has a distinct selective effect on electrogenetic bacteria.

Declaration of competing interest

The authors have declared no conflict of interest.
